# *Wolbachia* Infection in Native Populations of the Invasive Tawny Crazy Ant *Nylanderia fulva*

**DOI:** 10.3389/finsc.2022.905803

**Published:** 2022-06-06

**Authors:** María Belén Fernández, Christoph Bleidorn, Luis Alberto Calcaterra

**Affiliations:** ^1^ Fundación para el Estudio de Especies Invasivas (FuEDEI), Hurlingham, Argentina; ^2^ Consejo Nacional de Investigaciones Científicas y Técnicas (CONICET), Buenos Aires, Argentina; ^3^ Animal Evolution and Biodiversity, Johann-Friedrich-Blumenbach Institute for Zoology and Anthropology, Georg-August-University Göttingen, Göttingen, Germany

**Keywords:** *Wolbachia*, ants, invasive insects, horizontal transfer, phylogeny

## Abstract

Antagonistic interactions can affect population growth and dispersal of an invasive species. *Wolbachia* are intracellular endosymbiont bacteria that infect arthropod and nematode hosts and are able to manipulate reproduction, which in some cases leads to cocladogenesis. Moreover, the presence of the strictly maternally transferred *Wolbachia* in a population can indirectly induce selective sweeps on the hosts’ mitochondria. Ants have a *Wolbachia* infection rate of about 34%, which makes phylogenetic studies using mitochondrial markers vulnerable of being confounded by the effect of the endosymbiont. *Nylanderia fulva* is an invasive ant native to South America, considered a pest in the United States. Its distribution and biology are poorly known in its native range, and the taxonomic identity of this and its closely related species, *Nylanderia pubens*, has only recently been understood with the aid of molecular phylogenies. Aiming at estimating robust phylogenetic relationships of *N. fulva* in its native range, we investigated the presence and pattern of *Wolbachia* infection in populations of *N. fulva* from Argentina, part of its native range, to account for its possible effect on the host population structure. Using the *ftsZ* gene, 30 nests of *N. fulva* and four from sympatric *Nylanderia* species were screened for the presence of *Wolbachia*. We sequenced the MLST genes, the highly variable gene *wsp*, as well as *glyQ*, a novel target gene for which new primers were designed. Phylogeny of the ants was estimated using mtDNA (COI). We found supergroup A *Wolbachia* strains infecting 73% of *N. fulva* nests and two nests of *Nylanderia* sp. 1. *Wolbachia* phylogenetic tree inferred with MLST genes is partially congruent with the host phylogeny topology, with the exception of a lineage of strains shared by ants from different *N. fulva* clades. Furthermore, by comparing with *Wolbachia* sequences infecting other ants, we found that the strains infecting different *N. fulva* clades are not monophyletic. Our findings suggest there are three recent independent horizontally transmitted *Wolbachia* infections in *N. fulva*, and we found no evidence of influence of *Wolbachia* in the host mtDNA based phylogeny.

## Introduction

In the past few years, there has been increasing evidence of different endosymbiotic bacteria capable of affecting the biology of a variety of arthropod species, and most prominent are their effects on host reproduction (1). Among these organisms, the most common arthropod endosymbiont is *Wolbachia* (1). The *Wolbachia* genus encompasses a large phylogenetic diversity, with deeply diverging supergroups infecting arthropod and nematode hosts (2), but due to this enormous diversity within the genus, there is still some controversy on whether and how species names can be applied (e.g. 3, 4).

These bacteria ensure their proliferation within host populations by vertical transmission. Their effect on the host biology varies among host species and can include parthenogenesis, male feminization up to male-killing (5). The effects of these bacteria on their hosts can be detrimental, which has been of importance in some cases where *Wolbachia* was adopted as an agent to control pest insects or diseases transmitted by mosquitoes (6, 7). From an evolutionary perspective, *Wolbachia* has been documented to experience co-cladogenetic events with its host (e.g. 8) or to transfer fragments of its genome to its host (6). The most common process to enhance the spread of the strictly maternally transmitted *Wolbachia* in the population is the induction of cytoplasmic incompatibility (CI) (3), which can lead to selective sweeps that subsequently reduce mitochondrial DNA polymorphism in the host population (9). Their effect becomes more evident when closely related infected and uninfected taxa are compared (10).

Ants have an overall *Wolbachia* infection rate of about 34% (11), and effects of these endosymbionts can be alterations in colony life cycles, nutrition, and in production of reproductive individuals (12, 13). In the case of the invasive ant *Paratrechina longicornis*, it has been documented that the presence of maternally transmitted *Wolbachia* in a population can indirectly induce selective sweeps on the hosts’ mitochondria (14). As a consequence, *Wolbachia* may cause an accelerated spread in an invasive ant species due to an indirect selection of mitochondrial allelic variants that favor its invasive capacity.

*Nylanderia* is an ecologically important ant genus with a nearly cosmopolitan distribution. This genus was recently validated as the result of a reassessment of morphological characters complemented by a molecular phylogeny, and belongs to the *Prenolepis* genus-group (15). Many *Nylanderia* species that moved outside their native range became invasive (16). Such is the case of the tawny crazy ant, *Nylanderia fulva*, considered invasive in southern U.S. and native to South America, where the exact limits of its distribution are not well understood (17). Recent interest in the *Prenolepis* genus-group has provided with some important revisional studies about species status, re-descriptions and the discovery of species complexes. Complementing traditional morphological studies with genetic markers has helped understand the cryptic species complex that comprises *N. fulva* and *N. pubens* (18).

*Nylanderia fulva* is a species of omnivorous ant that forms polygynous nests that contain up to hundreds of reproductive queens and thousands of workers, which contributes to its capacity to quickly expand through its invasive range (16). It has been reported to reproduce sexually both in its native and invasive populations, but its social organization is multicolonial in the former and supercolonial in the latter (19, 20). Contrary to what has been documented for many other invasive ants (e.g. *Wasmannia auropunctata, Anoplolepis gracilipes, Paratrechina longicornis*), *N. fulva* does not present parthenogenetic reproduction neither in native nor invasive populations (19).

Traditionally, information from both mitochondrial and nuclear DNA can help understand the evolutionary history of a group of organisms at different taxonomic levels. Intraspecific variability in mitochondrial DNA (mtDNA) can be attributed to various possible evolutionary scenarios. Particularly, in *Wolbachia* infected species, the possibility of a selective sweep or some form of reproductive alteration can be expected when exploring phylogenies based on mtDNA data. In a review of the usage of mtDNA as a marker to infer phylogenies, the authors found in about 90% of the studies a symbiont-driven effect on the host mtDNA, such as a reduction of diversity or paraphyly (21).

*Wolbachia* detection and strain characterization has changed over time. For the past 15 years, the multi-*locus* sequence typing (MLST) system of five housekeeping gene fragments (*ftsZ*, *fbpA*, *gatB*, *hcpA* and *coxA*) has been the most popular method (22), lately, with the increased accessibility of next-generation sequencing, it has shifted also to whole-genome approaches (23). Bleidorn and Gerth (23) evaluated the reliability of MLST data compared to whole-genome data, to find that MLST *loci* and the highly recombinant *wsp* (*Wolbachia* surface protein) gene do not perform well at differentiating between closely related *Wolbachia* strains and also do not match the phylogenetic relationships seen by analyzing whole-genome data. However, the simplicity of using a reduced set of genes already established is still the most cost and/or time effective way to answer some questions compared to genomic approaches.

With the aim to evaluate if *N. fulva* phylogenetic relationships in its native range are influenced by *Wolbachia*, we first investigated the prevalence of infection and diversity of *Wolbachia* strains infecting *Nylanderia* species from northern Argentina, southern limit of its native range. Second, we investigated the relationship between the hosts mitochondrial DNA and its associated *Wolbachia* strains. Finally, we designed primers for the best ranked gene suggested by Bleidorn and Gerth (23) and evaluated how this gene fragment performed compared to the MLST approach.

## Methods

### *Nylanderia fulva* Mitochondrial DNA

Ants were manually collected from 35 nests throughout northeastern Argentina. Identification was done up to morphospecies, since updated keys for *Nylanderia* species are not available for the studied region, resulting in: 30, 2, 2 and 1 samples of *N. fulva*, *Nylanderia* sp. 1, *Nylanderia* sp. 2 and *P. longicornis*, respectively (**Table S1**). Genomic DNA was extracted from a single worker ant per nest using Extract-N-Amp Tissue PCR kit (Sigma-Aldrich Inc., St. Louis, MO, USA). Ant cytochrome C oxidase subunit I (COI) gene fragments were amplified by PCR using *Jerry* and *Pat primer* pair (18) and sequencing was performed by Macrogen (Macrogen Inc., Seoul, South Korea).

### Screening for *Wolbachia* Infection and Sequencing of MLST and *wsp* Genes

For detection of *Wolbachia* infection, genomic DNA was extracted from a single ant worker from each nest using GeneMATRIX Tissue & Bacterial DNA Purification Kit (EURx, Gdansk, Poland). To determine the infection status of the ants, 1-2 ants per nest were screened by PCR amplification of *ftsZ* gene. Some samples that did not amplify with *ftsZ* were cross-validated using *coxA*, which confirmed the same negative results in all cases (n=6). Positive samples were further amplified for the MLST genes described in Baldo et al. (22) and *wsp* gene, using *primers* and modified PCR protocols from Baldo et al. (22) (**Table S2**). Sequencing in both directions was provided by Microsynth (Microsynth Seqlab GmbH, Germany). Sequence typing was performed in the *Wolbachia* MLST database (https://pubmlst.org/bigsdb?db=pubmlst_wolbachia_seqdef).

For the sample N084, multiple peaks in the electropherogram profiles of genes *coxA, ftsZ, hcpA, wsp* and *glyQ* may indicate the presence of more than one *Wolbachia* variant. We used pGEM-T vector system (Promega) to clone these sample’s DNA fragments into a vector, and afterwards used primers previously described in this work to select colonies with the desired PCR insert. We obtained two variants for *coxA*, two for *glyQ*, one for *ftsZ* and one for *wsp* (cloning could not be achieved for *hcpA*). The *glyQ* variants are hereafter denoted N084a and N084b, and *coxA* variants N084c and N084d. Complete resolution of *ftsZ, hcpA* and *wsp* was not possible, so we present only one allele for each.

### Development of a New *Primer* Set for *Wolbachia glyQ* Gene

Bleidorn and Gerth (23) evaluated the performance of *Wolbachia* MLST markers compared with 252 other single copy *loci* at strain differentiation, reflecting genetic diversity in the strains and as phylogenetic markers for *Wolbachia*. They suggested a rated list of these *loci* from which *glycine-tRNA ligase subunit alpha* (*glyQ*) is the best ranked. We designed a new set of *primers* for this *locus* using Primer Premier v. 6.25 software (Premier Biosoft International, San Francisco, CA, USA) and a group of eleven available *Wolbachia* sequences from the dataset of Bleidorn and Gerth (23): *glyQF* (forward) 5’-GCAATGGAATGGAAGTAACACAG-3’ and *glyQR* (reverse) 5’-YTCACACCAAGCACACCTCT-3’. We selected from the potential primer pairs according to their Tm, self- and cross-dimer formation, and potential to form secondary structure (hairpins). Sequences were obtained using the same PCR protocols as for other MLST *loci* (**Table S1**), and aligned as previously stated. Blast algorithm (https://blast.ncbi.nlm.nih.gov/Blast.cgi) was used to corroborate that the sequenced fragments belonged to *Wolbachia.* Additional sequences were obtained from *Wolbachia* genomes deposited in Genbank (**Table S3**).

### Phylogenetic Relationships

Chromatograms of the sequences were visually inspected in Chromas v2.6.6 (Technelysium Pty Ltd, Australia). Alignment was done in MEGA X (24) with the Muscle algorithm (25) using the default parameters for both *Wolbachia* and *Nylanderia* spp. data. DNA sequence diversity estimates were calculated in DnaSP v6 (26). Additional sequences from repositories were included as outgroups, for determination of supergroup identity and diversity of the *Wolbachia* samples (**Table S3**). Two gene sets were used to estimate *Wolbachia* trees: MLST *loci* (*ftsZ, coxA, fbpA, hcpA and gatB*) (2079 bp), and *glyQ* (333 bp). *Wsp* gene was not used to infer phylogenies due to its high recombination rate. Selection of the best-fitting evolutionary model, maximum likelihood (ML) analyses and bootstrapping (1000 replicates) were performed for all datasets with IQ-TREE v. 2 (27, 28). Finally, to evaluate the potential of the selected fragment of *glyQ* gene, we compared the phylogeny of *Wolbachia* based on MLST *loci* with one estimated with *glyQ* (**Figure S1**).

## Results

**Figure 1:**
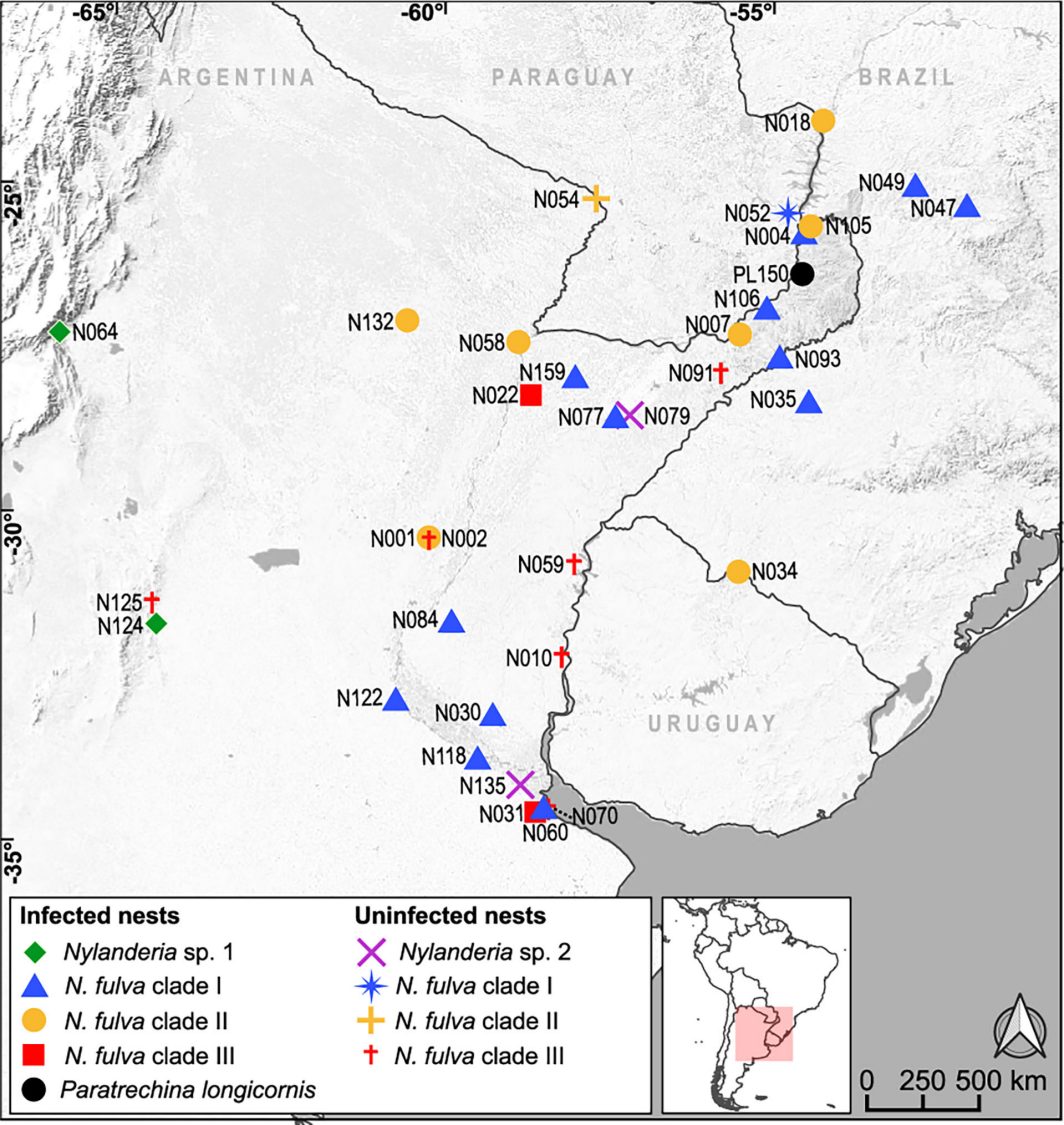
Geographic location of the nests of *Nylanderia* sp.1, *Nylamderia* sp. 2, *Nylanderia fulva* and *Paratrechina longicornis* infected and uninfected with *Wolbachia*. Colors in *N. fulva* correspond to the clades determined in this work (**Figure 2**).

After inspection of the presence of the *Wolbachia ftsZ* gene, we found infections in 41% of the 34 *Nylanderia* nests, and a high percentage of infected nests within *N. fulva* (68%) (**Table 1**). Distribution of the *Wolbachia* carrying nests spanned throughout northeastern Argentina (**Figure 1**). The sample of *P. longicornis* (PL150), resulted positive for *Wolbachia*, as well as *Nylanderia* sp. 1 with both nests infected. *Nylanderia* sp. 2 had no positive nests for *Wolbachia*.

**Table 1:** Prevalence of *Wolbachia* infection in *Nylanderia* spp. One to two workers per nest were tested for presence of *Wolbachia*. Clades are based on the ants’ phylogeny from this work (**Figure 2**).

		Infected	Uninfected
***Nylanderia fulva***	**clade I**	13 (93%)	1
	**clade II**	6 (75%)	2
	**clade III**	2 (25%)	6
***Nylanderia* sp. 1**		2 (100%)	0
***Nylanderia* sp. 2**		0 (0%)	2
**Total**		23 (68%)	11

**Figure 2:**
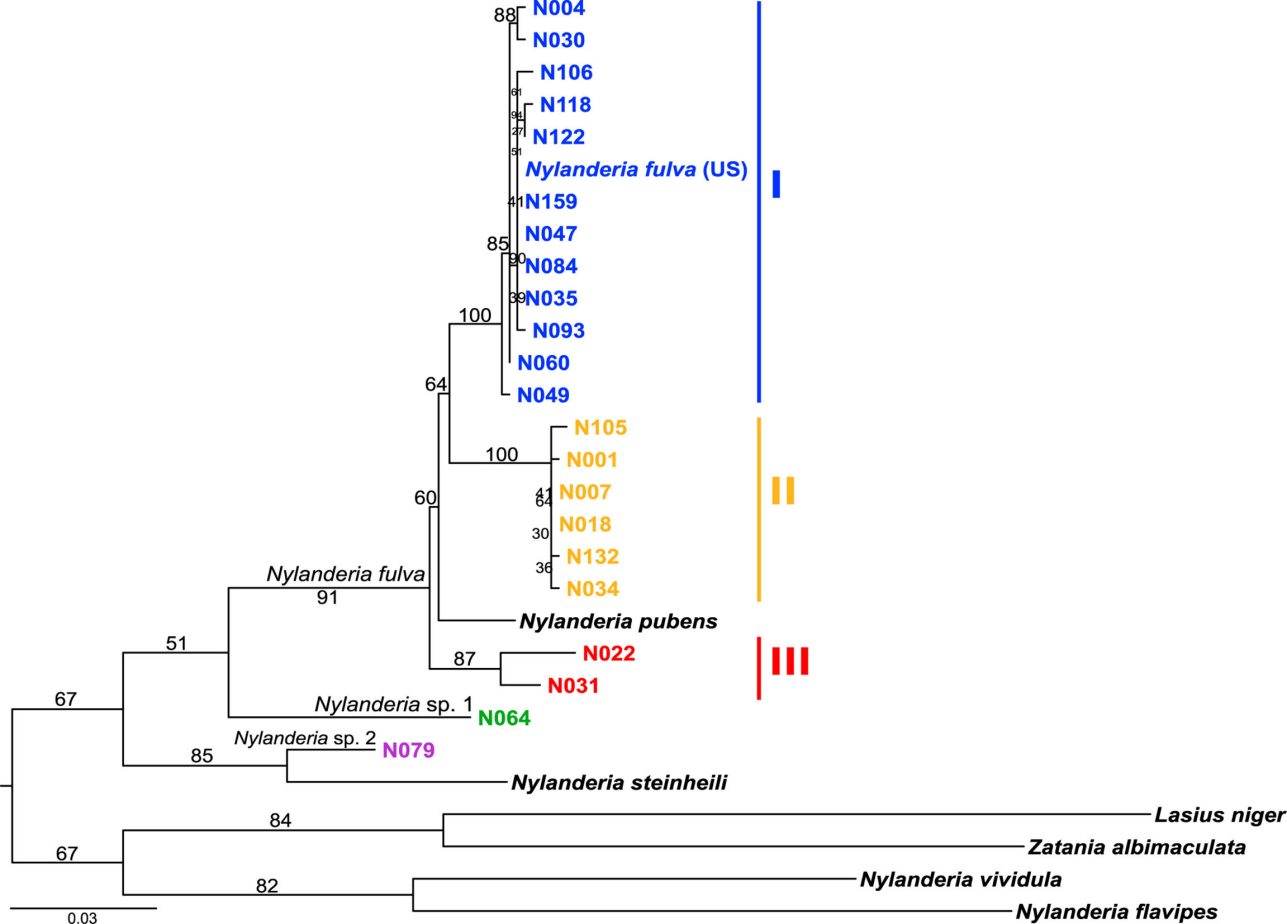
*Nylanderia fulva* maximum likelihood phylogeny based on mtDNA gene COI. Monophyletic lineages within *N. fulva* are color coded: clade I (blue), II (yellow) and III (red).

Phylogenetic relationships of *Nylanderia* spp. were estimated using a fragment of the mitochondrial gene COI (**Figure 2**). *Nylanderia fulva* was recovered as paraphyletic, with *N. pubens* as a sister species. Within *N. fulva*, we differentiate three monophyletic clades, which were identified as clades I, II and III. The sequence belonging to an invasive population found in the US falls within clade I of *N. fulva*. Nucleotide diversity is lower in clades I and II, than in clade III (0.0035, 0.0024, and 0.0203, respectively), although sampling size for the latter was smaller. We tested the hypothesis of a selective sweep in mitochondrial DNA of *N. fulva* clades I and II (clade III was excluded due to its small sample size). All statistical tests of departure from neutral expectation were negative in both clades I and II (Tajima’s D, Fu and Li’s D* and F*, **Table S4**), but not significant. *Wolbachia* infection status was different for each *N. fulva* clade: clade I had the highest number of infected nests (93%), clade II had 75%, and clade III had the lowest infection rate with only 25% of positive nests (**Table 1**). *Nylanderia* sp. 1 was recovered as a sister species to *N. fulva*. Phylogenetic position of *N. fulva* and *N*. sp. 1 within other *Nylanderia* species is uncertain, due to the low support value of this branch, but both are closely related to another invasive species, *Nylanderia steinheili*.

Almost all of the *Wolbachia* infected samples could be successfully sequenced for the five *Wolbachia* MLST genes, with exception of *gatB*, which could only be amplified for three samples (N031, N060, and PL150). For sequencing with the pair of primers for the *glycine-tRNA ligase subunit alpha* (*glyQ*) gene, we implemented similar PCR protocols as stated in *Wolbachia* MLST database and successfully amplified all of the infected samples for a fragment of 333 bp.

By comparing these sequences with published *Wolbachia* MLST strain database (https://pubmlst.org/organisms/wolbachia-spp/), we determined the sequence type (ST) for our samples, when possible, and defined strain names for 13 unique MLST profiles, *w*Nyla1 to *w*Nyla13 (**Table 2**). Within *N. fulva* clade I nests, we found the highest number of *Wolbachia* strains (eight), while in clades II and III there are only two different strains infecting each. One of the *Wolbachia* strains infecting *N. fulva* clade II, *w*Nyla1, is highly prevalent, infecting six out of seven inspected nests. In *Nylanderia* sp. 1, the same *Wolbachia* strain infects both nests. We would like to point out, however, that since *gatB* sequences are lacking for most of the samples, different results are possible and could show higher variability. We found no identical sequence variants infecting different *Nylanderia* clades. Variability in *wsp* gene was high, with eight different alleles found in *Nylanderia* spp., from which two are novel. Diversity estimates for the sequenced gene fragments show that the most variable gene was *ftsZ*, presenting eleven different haplotypes, and the highest nucleotide diversity was found in *wsp* gene (**Table 3**). Compared with the MLST *loci* all together, the *glyQ* fragment had an overall low diversity, with only five different haplotypes for *Nylanderia* spp. and only nine segregating sites. However, the fragment presents similar percentage of segregating sites (about 3%) and nucleotide diversity (0.01) to other MLST genes alone, such as *coxA* and *ftsZ*. Additionally, if *glyQ* gene is considered when establishing sequence types, the number of different haplotypes within *Nylanderia* spp. rises from 13 to 14 (**Table S5**).

**Table 2:** Allelic profiles, *wsp* and HVR allele number identifiers for *Wolbachia* strains in *Nylanderia* ants.

Host species (clade)	Sample code	Strain Name (MLST)	*gatB*	*coxA*	*hcpA*	*ftsZ*	*fbpA*	*wsp*	HVR1	HVR2	HVR3	HVR4
***Nylanderia fulva* (clade I)**	N004	*w*Nyla2	–	15	i	6	1	408	9	9	12	271
	N030	*w*Nyla4	–	15	i	i	1	408	9	9	12	271
	N035	*w*Nyla6	–	20	45	ii	–	351	21	21	25	317
	N047	*w*Nyla7	–	15	i	iii	1	408	9	9	12	271
	N049	*w*Nyla7	–	15	i	iii	1	408	9	9	12	271
	N060	*w*Nyla6	43	20	45	ii	–	351	21	21	25	317
	N077	*w*Nyla7	–	15	i	iii	1	408	9	9	12	271
	N084	*w*Nyla8 *w*Nyla9	– –	15 20	iii iii	ii ii	1 1	351 351	21 21	21 21	25 25	317 317
	N093	*w*Nyla10	–	15	i	iv	1	ii	9	9	12	130
	N106	*w*Nyla7	–	15	i	iii	1	408	9	9	12	271
	N118	*w*Nyla12	–	ii	i	iii	1	408	9	9	12	271
	N122	*w*Nyla7	–	15	i	iii	1	408	9	9	12	271
	N159	*w*Nyla13	–	20	45	vi	–	351	21	21	25	317
***N. fulva* (clade II)**	N001	*w*Nyla1	–	20	44	37	i	58	37	38	41	317
	N007	*w*Nyla1	–	20	44	37	i	58	37	38	41	317
	N018	*w*Nyla1	–	20	44	37	i	58	37	38	41	317
	N034	*w*Nyla1	–	20	44	37	i	58	37	38	41	317
	N058	*w*Nyla1	–	20	44	37	i	58	37	38	41	317
	N105	*w*Nyla11	–	20	45	v	–	351	21	21	25	317
	N132	*w*Nyla1	–	20	44	37	i	58	37	38	41	317
***N. fulva* (clade III)**	N022	*w*Nyla3	–	20	ii	17	20	50	42	43	9	272
	N031	*w*Nyla5	43	i	45	ii	–	i	21	21	25	317
***Nylanderia* sp. 1**	N064	*w*Nyla14	–	iii	iv	vii	ii	iii	9	9	12	272
	N124	*w*Nyla14	–	iii	iv	viii	ii	iii	9	9	12	272
***Paratrechina longicornis***	PL150	ST471	168	147	262	132	226	708	242	274	276	308

Roman numbers indicate unique alleles not found in Wolbachia MLST database. Strain names are given for each unique combination of MLST loci, and the sequence type (ST) for the sample PL150. Sample N084 was co-infected with two similar Wolbachia strains (wNyla8 and wNyla9).

**Table 3:** DNA variation estimates for each sequenced *Wolbachia* gene in *Nylanderia* spp. infected ants.

	*gatB*	*coxA*	*hcpA*	*ftsZ*	*fbpA*	MLST	*wsp*	*glyQ*
**n**	2	24	23	24	19	24	24	24
**H**	2	5	6	11	4	13	7	5
**L**	369	402	444	435	429	2079	508	333
**s**	–	11	31	14	32	56	140	9
**π**	–	0.00914	0.02375	0.01032	0.03353	0.01463	0.09275	0.01014

n, sequence number; H, haplotype number; L, sequence length in base pairs; s, number of segregating sites; π, nucleotide diversity.

**Figure 3:**
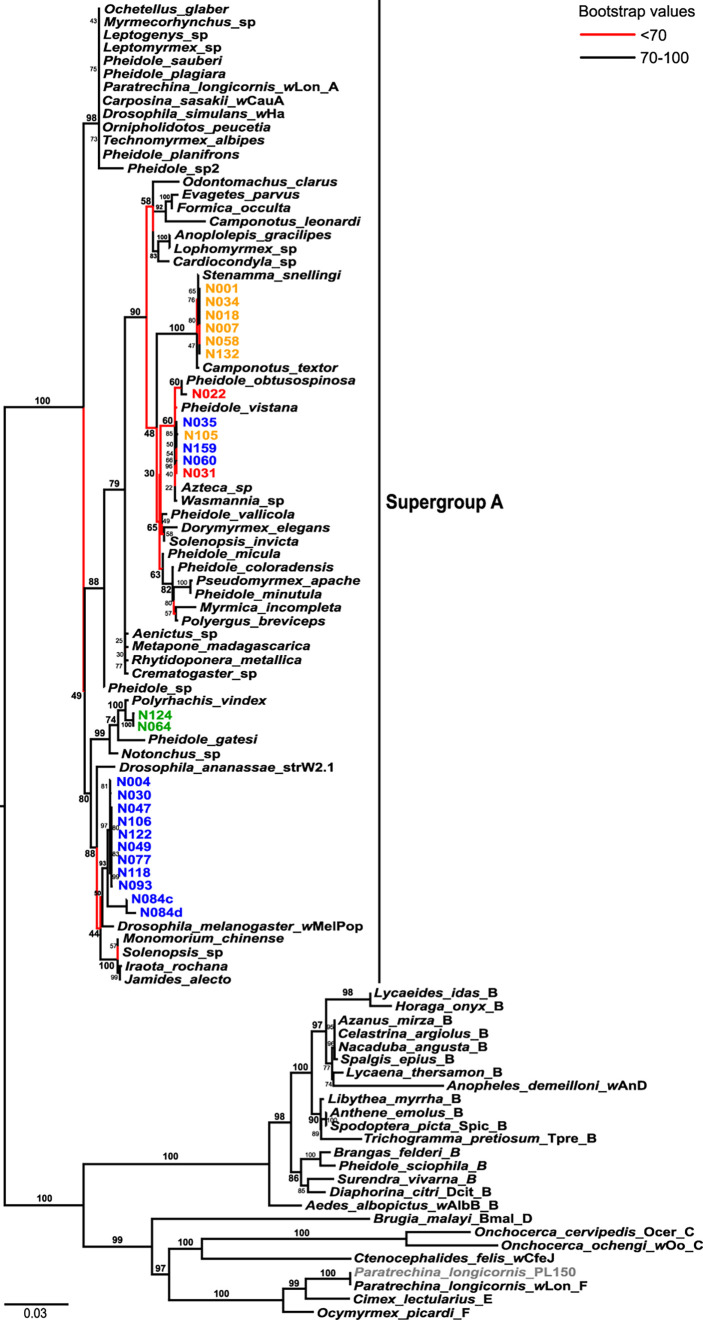
*Wolbachia* maximum likelihood phylogeny based on multi locus sequence typing (MLST) data with host species as labels. Colors highlight the sequences produced in this work belonging to different *Nylanderia* mitochondrial DNA clades: clade I (blue), clade II (yellow), clade III (red), and *Nylanderia* sp. 1 (green). All sequences belong to *Wolbachia* supergroup A unless stated with its corresponding letter (B-F). Branches with bootstrap support values lower than 70 are indicated in red.

In the MLST phylogeny of *Wolbachia* strains infecting *Nylanderia* spp., all of the strains belong to supergroup A, while *P. longicornis* was infected with the strain *w*LonF (ST471), belonging to supergroup F (**Figure 3**). Most nests of *N. fulva* clades I and II are infected each with a unique lineage of *Wolbachia* strains, while clade III harbors two distantly related *Wolbachia* strains. There is one group of *N. fulva* nests belonging to clades I, II and III and from various geographic locations that share infection of the same lineage of *Wolbachia* strains (samples N031, N035, N060, N105 and N159). The four *Wolbachia* lineages infecting *N. fulva* are distantly related with each other and do not form a single monophyletic unit. *Nylanderia* sp. 1 is also infected with a unique *Wolbachia* strain that is closely related to those infecting other ants.

The *Wolbachia* phylogeny inferred with the *glyQ* fragment shows a similar pattern as that of the MLST tree (**Figure S1**). *Nylanderia* fulva clades appear infected by three monophyletic *Wolbachia* lineages: one infecting *N. fulva* clade I nests, another infecting clade II nests, and the third infecting nests that belong to clades I, II and III. On the other hand, these lineages show a different relationship with each other as that seen in the MLST tree. *Nylanderia* sp. 1 *Wolbachia* strain is more closely related to that infecting *N. fulva* clade II in the *glyQ* phylogeny, but it is more related to clade I when considering the MLST dataset. Plus, comparison with sequences of *Wolbachia* infecting other insects is limited by available complete genomes, and thus, limits the possibility to compare the relationship of the strain lineages with those of other hosts. Variants found in the sample N084 were distantly related, with variant N084a closely related to those infecting mostly clade I ants, while variant N084b is placed within the group of *Wolbachia* infecting samples from every *N. fulva* clade.

## Discussion

### *Nylanderia* Phylogeny

We investigated the phylogenetic relationships of an invasive ant species, *Nylanderia fulva*, by sampling nests across the southern limit of its native distribution. Using the mitochondrial DNA marker COI, we found that *N*. *fulva* was conformed of three distinct monophyletic clades (clades I to III) from which clade I included an invasive population found in the US. We proposed a possible *Wolbachia* influence on *N. fulva* population structure based on the pattern of reciprocal monophyletic lineages observed in mtDNA. A wide range of causes can explain this observation, such as a speciation process, a recent population expansion or a selective sweep, or a combination of processes affecting each clade differently.

In addition, *N. pubens* was placed within the boundaries of what we considered the species *N. fulva*, and thus supports its position as a sibling species. This is in line with a recent work suggesting that *N. fulva* and *N. pubens* form a species complex (29). These two species have been commonly mistaken due to the identical aspect of their workers, but are distinguishable inspecting male genital characters (18). More sampling of *N. fulva* and *N. pubens* throughout their native ranges with a detailed examination of male genitalia should provide useful evidence to understand the limits of these two close species.

Furthermore, we found another two *Nylanderia* species present in the surveyed region. *Nylanderia* sp. 1, which we consider could be the only other common *Nylanderia* species historically reported for the region, *Nylanderia silvestrii* (30), and *N.* sp. 2, which possibly belongs to the *N. guatemalensis/N. steinheili* species complex. This would be a novel location record for this invasive species in Argentina, in Corrientes province, being previously recorded in the city of Buenos Aires (31). Further analyses of morphological characters and sequencing of type material could be of aid in confirming the identity of these species.

### *Wolbachia* Infection and Diversity in *Nylanderia* spp.

We have detected *Wolbachia* infection for the first time in the invasive ant *Nylanderia fulva* in its native range. Additionally, infection of this endosymbiont was found in other two species, *N*. sp. 1, and the closely related invasive ant species, *P. longicornis*. Strains infecting *Nylanderia* species belonged to supergroup A, while that of *P. longicornis* was identified as *w*LonF, from supergroup F. These are the first records of *Wolbachia* infection for the three species in this region, as well as the first confirmed record of invasion of *P. longicornis* in Argentina. The strain infecting *P. longicornis*, *w*LonF, is primarily horizontally transmitted and widely distributed in this ant around the globe (14). *Wolbachia* infecting ants mostly belong to supergroup A, secondly to B and a few exceptions to supergroup F, namely *Paratrechina longicornis* and *Ocymyrmex picardi* (32, 33). We found that all *Wolbachia* infecting *Nylanderia* species belonged to supergroup A, which is consistent with those infecting most of other insect hosts, particularly ants (12). We found no infection of *Wolbachia* in *Nylanderia* sp. 2, a putatively invasive species belonging to the *N. guatemalensis/N. steinheili* species complex. This result coincides with that seen for other invasive ant species, whose introduced populations are usually uninfected (e.g. 34, 35). Furthermore, we found no distinctive distributional pattern of infected/uninfected *Nylanderia* nests in the surveyed area; *Wolbachia* infected nests were found throughout northeastern Argentina, southeastern Brazil and in some locations of Paraguay and Uruguay.

Several MLST alleles for the infected *Nylanderia* samples did not match those in the *Wolbachia* MLST database. We reported 14 new MLST sequence types, which include 17 new alleles: three for *coxA*, four for *hcpA*, eight for *ftsZ*, and two for *fbpA*. Additionally, we found two new alleles for the *wsp* gene to be uploaded to the MLST database.

We designed a new set of primers for a fragment of the *glycine-tRNA ligase subunit alpha* (*glyQ*) gene and a PCR protocol that is readily available for use. This fragment presented five different haplotypes for the sampled *Wolbachia* strains, and overall it matches diversity estimates of other widely used gene fragments such as *coxA* and *ftsZ*. When combined with the MLST *loci*, we retrieved one additional haplotype or sequence type, for a total of 15 different haplotypes in *Nylanderia* spp. This suggests there is at least some degree of variation in *Wolbachia* strains still not uncovered by the MLST system, at least for the evaluated species. With the availability of assemblies of more than 20 *Wolbachia* genomes, more and more information become available to perform genome-wide *Wolbachia* phylogenies. Chung et al. (4) found more *Wolbachia* species using a core genome alignment than established supergroups. In other bacteria, such as *Staphylococcus aureus* or *Haemophilus influenzae*, the MLST system is comprised of more than five genes, so the incorporation of a new gene could be an interesting addition for *Wolbachia* MLST scheme (36, 37). Furthermore, given that we were not able to amplify most samples with one of the MLST genes, *gatB*, and that the protocols and primers established here for *glyQ* were successful, the possibility to include more genes to the MLST system can be useful in these cases for a more thorough determination of *Wolbachia* haplotypes.

Though our results show a high rate of *Wolbachia* infection within some lineages of *N. fulva*, there is a limitation to our findings regarding the small number of ants sampled by nest. Diversity estimates as well as prevalence values should be taken as a first approximation since more individuals per nest should be inspected to fully understand the prevalence of *Wolbachia* within populations.

### Incongruence Between *Wolbachia* and *Nylanderia fulva* Phylogenies

We explored the relationship of *Wolbachia* strains infecting *N. fulva* according to their three host clades, clades I to III. Three *Wolbachia* lineages were found infecting *N. fulva* clades, although with different prevalence; clade I had the most infected number of nests, second was clade II and clade III had the lowest number of infected nests. We found low nucleotide diversity in *N. fulva* clades I and II, which can be related to a putative population expansion or population bottleneck, but also to recent symbiont invasions, where selective sweeps cause lower mtDNA diversity, and so can easily be mistaken for the effects of the former events (21). In other insect species with partial *Wolbachia* infection, as was the case in *N. fulva*, mitochondrial polymorphism tends to be lower in the infected lineages; additionally, in sister species, *Wolbachia* infection has been related to reduction in the effective population size (10). Recent studies suggest *Wolbachia* could cause speciation in its host *via* induced parthenogenesis; for example, in the weevil *Pantomorus postfasciatus*, all non-sexual populations harbor *Wolbachia* while the sexual ones are not infected, and both behave as independent evolutionary units (38). In the invasive ant *P. longicornis*, it was suggested that an ancestral *Wolbachia* infection could be associated with a recent speciation in the ants’ clades (8, 14). High prevalence of *Wolbachia* within clade I may imply an intimate association between the symbiont and its host, but empirical tests would be needed to explore this relationship. Furthermore, Eyer et al. (19) found no evidence of parthenogenetic reproduction in *N. fulva.* We found no evidence of departure from neutrality in all the tested statistics within clades I or II that could be associated with a selective sweep, although values for the statistics were negative, which can be related to a selection process. We do not fully discard the possibility of a *Wolbachia* induced selective sweep due to the low sampling size within nests that can underestimate intraspecific variability. There were no clades of *N. fulva* completely uninfected with *Wolbachia*, as was the case in other species. Altogether, these results compete with the hypothesis of a selective sweep affecting one particular clade. It has been hypothesized that horizontal transfer as well as a degree of admixture between sexual and parthenogenetic populations, can alter the effects of a vertically transmitted strain, resulting in a hindered *Wolbachia* fixation and, hence, obscuring the effects of the selective sweep (2). Reproductive isolation experiments could provide useful information to validate this possibility.

The pattern of monophyletic lineages of *Wolbachia* infecting different groups of *N. fulva* is incongruent with that observed in the host phylogeny. While in the host phylogeny the three *N. fulva* clades are closely related and form a monophyletic unit (together with *N. pubens*), in the *Wolbachia* phylogeny the lineages are paraphyletic. Also, some of the *N. fulva* nests belonging to different clades are infected with the same lineage of *Wolbachia* strains. There are some cases, such as that of *Culex pipiens*, in that identical strains based on MLST sequence types (STs) and *wsp* alleles in fact present differences in other inspected genes and significant structural variation that in turn produce different phenotypic effects (39). All of the *Wolbachia* lineages found in *Nylanderia* nests are closely related to strains found infecting other insects, such as other ants and *Drosophila* flies. In light of these results, the discordance between phylogenies of *Wolbachia* and *N. fulva* suggests that infection mechanisms of *Wolbachia* could involve various independent horizontally transmitted *Wolbachia* infections within *N. fulva*. Similar results have been obtained for another invasive species, the little fire ant *Wasmannia auropunctata* (40), who’s native distribution range also overlaps with that of *N. fulva*. Close relationship of the different *Wolbachia* infecting *Nylanderia* with other insects suggests horizontal transmission (HT), possibly through sharing of some aspect of their ecological niches. There are many ways in which *Wolbachia* can be horizontally transferred between closely related species, for example, if they share parasitoids or parasites. Parasitic phorid flies have been considered a potential source of HT in fire ants, *Solenopsis* spp., but there is yet no data supporting a shared *Wolbachia* strain with their hosts (41, 42). The only phorid fly species reported parasitizing *N. fulva* up to now*, Pseudacteon convexicauda* (43), could be acting as a vector of *Wolbachia* between *N. fulva* clades, thus facilitating its transmission. Ant guests can also present an opportunity for *Wolbachia* to be horizontally transmitted to the ants, as was the case for *P. longicornis* and its host-specific ant cricket *Myrmecophilus americanus*; although in the invasive ant *Anoplolepis gracilipes*, HT was not present between the ants and their associated cricket species *M. albicinctus* (8). Moreover, other relationships, such as prey-predator interactions, can also be a way of horizontal transmission (1, 7, 44). Ant workers interact directly with brood by tending and feeding them and frequently with sister workers through trophallaxis, a mechanism in which workers transfer food or other fluids through mouth-to-mouth (45). These interactions could be a means or perpetrating a horizontally transmitted strain within the nest.

An invasive population of *N. fulva* in the US was found to be closely related to native populations from Argentina belonging to clade I. Native populations of *N. fulva* clade I showed the highest prevalence of infection and number of *Wolbachia* strains. In another invasive ant sympatric with *N. fulva* in Argentina, *Solenopsis invicta*, it was suggested that possible loss of *Wolbachia* infection in invasive populations may have a relationship with its invasiveness, and that re-introduction of *Wolbachia* might be a means of biological control of the ants, if its effect is indeed deleterious (11, 34). Data from invasive populations is needed to confirm if they are infected with *Wolbachia*.

We found no correlation in phylogenetic relationships of *Wolbachia* strains infecting *N. fulva* and the host mtDNA phylogeny. Our research suggests that horizontal transmission could be the most prominent way of *Wolbachia* transmission in these ants, and that there is no evidence that supports an influence of *Wolbachia* in *Nylanderia* mtDNA phylogeny. However, it could be interesting to further investigate prevalence of *Wolbachia* infection in the introduced *N. fulva* populations and in other *Nylanderia* species due to the small sampling size included in this study for *Nylanderia* sp. 1 and *Nylanderia* sp. 2. By incorporating more genetic markers, such as nuclear DNA for the ants, and larger sampling, it may be possible to reveal even more variability than we observed in this work, and to have a better understanding of the patterns and consequences of *Wolbachia* infection in these ants.

## Data Availability Statement

The dataset containing sequence alignments used in this paper can be found in Zenodo repository under the DOI address: 10.5281/zenodo.6570990.

## Author Contributions

MBF and LC collected the samples. MBF performed the experiments and analyses. MBF, LC, and CB analyzed the data and contributed to writing the manuscript. All authors contributed to the article and approved the submitted version.

## Funding

MBF was funded by fellowships from Consejo Nacional de Investigaciones Científicas y Técnicas (CONICET), Universidad de Buenos Aires (UBA) and Potsdam University.

## Conflict of Interest

The authors declare that the research was conducted in the absence of any commercial or financial relationships that could be construed as a potential conflict of interest.

## Publisher’s Note

All claims expressed in this article are solely those of the authors and do not necessarily represent those of their affiliated organizations, or those of the publisher, the editors and the reviewers. Any product that may be evaluated in this article, or claim that may be made by its manufacturer, is not guaranteed or endorsed by the publisher.

## References

[1] Duron O Bouchon D Boutin S Bellamy L Zhou L Engelstädter J. The Diversity of Reproductive Parasites Among Arthropods: *Wolbachia* Do Not Walk Alone. BMC Biol (2008) 6:1–12. doi: 10.1186/1741-7007-6-27 18577218 PMC2492848

[2] Kaur R Shropshire JD Cross KL Leigh B Mansueto AJ Stewart V. Living in the Endosymbiotic World of *Wolbachia*: A Centennial Review. Cell Host Microbe (2021) 29(6):879–93. doi: 10.1016/j.chom.2021.03.006 PMC8192442 33945798

[3] Werren JH Baldo L Clark ME. *Wolbachia*: Master Manipulators of Invertebrate Biology. Nat Rev Microbiol (2008) 6(10):741–51. doi: 10.1038/nrmicro1969 18794912

[4] Chung M Munro JB Tettelin H Dunning Hotopp JC. Using Core Genome Alignments to Assign Bacterial Species. mSystems (2018) 3:e00236–18. doi: 10.1128/mSystems.00236-18 PMC6280431 30534598

[5] Asimakis ED Doudoumis V Hadapad AB Hire RS Batargias C Niu C. Detection and Characterization of Bacterial Endosymbionts in Southeast Asian Tephritid Fruit Fly Populations. BMC Microbiol (2019) 19(Suppl 1):1–18. doi: 10.1186/s12866-019-1653-x 31870298 PMC7050614

[6] Rodriguero MS. Wolbachia, a Pandemic With Potential. In: Revista De La Sociedad Entomológica Argentina. (2013), 117–37.

[7] Sanaei E Charlat S Engelstädter J. *Wolbachia* Host Shifts: Routes, Mechanisms, Constraints and Evolutionary Consequences. Biol Rev (2021) 96(2): 433–53. doi: 10.1111/brv.12663 33128345

[8] Lee CC Lin CY Tseng SP Matsuura K Yang CCS. Ongoing Coevolution of *Wolbachia* and a Widespread Invasive Ant, *Anoplolepis Gracilipes* . Microorganisms (2020) 8(10):1–17. doi: 10.3390/microorganisms8101569 PMC7601630 33053771

[9] Schuler H Köppler K Daxböck-Horvath S Rasool B Krumböck S Schwarz D. The Hitchhiker’s Guide to Europe: The Infection Dynamics of an Ongoing *Wolbachia* Invasion and Mitochondrial Selective Sweep in *Rhagoletis Cerasi* . Mol Ecol (2016) 25(7):1595–609. doi: 10.1111/mec.13571 PMC4950298 26846713

[10] Cariou M Duret L Charlat S. The Global Impact of Wolbachia on Mitochondrial Diversity and Evolution. J Evol Biol (2017) 30:2204–10. doi: 10.1111/jeb.13186 28977708

[11] Russell JA. The Ants (Hymenoptera: Formicidae) are Unique and Enigmatic Hosts of Prevalent *Wolbachia* (Alphaproteobacteria) Symbionts. Myrmecol News (2012) 16(January):7–23.

[12] Russell JA Goldman-Huertas B Moreau CS Baldo L Stahlhut JK Werren JH. Specialization and Geographic Isolation Among *Wolbachia* Symbionts From Ants and Lycaenid Butterflies. Evolution (2009) 63(3):624–40. doi: 10.1111/j.1558-5646.2008.00579.x 19054050

[13] Ramalho MO Moreau CS. The Evolution and Biogeography of *Wolbachia* in Ants (Hymenoptera: Formicidae). Diversity (2020) 12(11):1–12. doi: 10.3390/d12110426

[14] Tseng SP Wetterer JK Suarez AV Lee CY Yoshimura T Shoemaker DW. Genetic Diversity and *Wolbachia* Infection Patterns in a Globally Distributed Invasive Ant. Front Genet (2019) 10:838. doi: 10.3389/fgene.2019.00838 31608104 PMC6758599

[15] LaPolla JS Brady SG Shattuck SO. Phylogeny and Taxonomy of the *Prenolepis* Genus-Group of Ants (Hymenoptera: Formicidae). Systematic Entomol (2010) 35(1):118–31. doi: 10.1111/j.1365.3113.2009.00492.x

[16] Williams JL Lucky A. Non-Native and Invasive *Nylanderia* Crazy Ants (Hymenoptera: Formicidae) of the World: Integrating Genomics to Enhance Taxonomic Preparedness. Ann Entomol Soc America (2019) 113(4):318–36. doi: 10.1093/aesa/saz039

[17] Wang Z Moshman L Kraus EC Wilson BE Acharya N Diaz R. A Review of the Tawny Crazy Ant, *Nylanderia Fulva*, an Emergent Ant Invader in the Southern United States: Is Biological Control a Feasible Management Option? Insects (2016) 7(4):77. doi: 10.3390/insects7040077 27983690 PMC5198225

[18] Gotzek D Brady SG Kallal RJ LaPolla JS. The Importance of Using Multiple Approaches for Identifying Emerging Invasive Species: The Case of the Rasberry Crazy Ant in the United States. PloS One (2012) 7(9):1–10. doi: 10.1371/journal.pone.0045314 PMC3462614 23056657

[19] Eyer PA McDowell B Johnson LNL Calcaterra LA Fernandez MB Shoemaker D. Supercolonial Structure of Invasive Populations of the Tawny Crazy Ant *Nylanderia Fulva* in the US. BMC Evol Biol (2018) 18(1):2007. doi: 10.1186/s12862-018-1336-5 PMC6310932 30594137

[20] LeBrun EG Plowes RM Folgarait PJ Bolazzi M Gilbert LE. Ritualized Aggressive Behavior Reveals Distinct Social Structures in Native and Introduced Range Tawny Crazy Ants. PloS One (2019) 14(11):e0225597. doi: 10.1371/journal.pone.0225597 31756233 PMC6874334

[21] Hurst GDD Jiggins FM. Problems With Mitochondrial DNA as a Marker in Population, Phylogeographic and Phylogenetic Studies: The Effects of Inherited Symbionts. Proc R Soc B: Biol Sci (2005) 272(1572):1525–34. doi: 10.1098/rspb.2005.3056 PMC1559843 16048766

[22] Baldo L Hotopp JCD Jolley KA Bordenstein SR Biber SA Choudhury RR. Multilocus Sequence Typing System for the Endosymbiont *Wolbachia* Pipientis. Appl Environ Microbiol (2006) 72(11):7098–110. doi: 10.1128/AEM.00731-06 PMC1636189 16936055

[23] Bleidorn C Gerth M. A Critical Re-Evaluation of Multilocus Sequence Typing (MLST) Efforts in *Wolbachia* . FEMS Microbiol Ecol (2018) 94(1):1–11. doi: 10.1093/femsec/fix163 29186405

[24] Kumar S Stecher G Li M Knyaz C Tamura K. MEGA X: Molecular Evolutionary Genetics Analysis Across Computing Platforms. Mol Biol Evol (2018) 35:1547–9. doi: 10.1093/molbev/msy096 PMC5967553 29722887

[25] Edgar RC. MUSCLE: Multiple Sequence Alignment With High Accuracy and High Throughput. Nucleic Acids Res (2004) 32(5):1792–7. doi: 10.1093/nar/gkh340 PMC390337 15034147

[26] Rozas J Ferrer-Mata A Sanchez-DelBarrio JC Guirao-Rico S Librado P Ramos-Onsins SE. DnaSP V6: DNA Sequence Polymorphism Analysis of Large Datasets. Mol Biol Evol (2017) 34:3299–302. doi: 10.1093/molbev/msx248 29029172

[27] Kalyaanamoorthy S Minh BQ Wong TKF von Haeseler A Jermiin LS. ModelFinder: Fast Model Selection for Accurate Phylogenetic Estimates. Nat Methods (2017) 14:587–9. doi: 10.1038/nmeth.4285 PMC5453245 28481363

[28] Minh BQ Schmidt HA Chernomor O Schrempf D Woodhams MD von Haeseler A. IQ-TREE 2: New Models and Efficient Methods for Phylogenetic Inference in the Genomic Era. Mol Biol Evol (2020) 37:1530–4. doi: 10.1093/molbev/msaa015 PMC7182206 32011700

[29] Williams JL Zhang YM Lloyd MW LaPolla JS Schultz TR Lucky A. Global Domination by Crazy Ants: Phylogenomics Reveals Biogeographical History and Invasive Species Relationships in the Genus *Nylanderia* (Hymenoptera: Formicidae). Systematic Entomol (2020) 45:730–44. doi: 10.1111/syen.12423

[30] Kusnezov N. Hormigas Argentinas: Clave Para Su Identificacion. Golbach R, editor. Tucumán, Argentina: Fundacion Miguel Lillo (1978).

[31] Josens R Sola FJ Marchisio N Di Rienzo MA Giacometti A. Knowing the Enemy: Ant Behavior and Control in a Pediatric Hospital of Buenos Aires. SpringerPlus (2014) 3:229. doi: 10.1186/2193-1801-3-229 24855592 PMC4024480

[32] Kelly M Price SL de Oliveira Ramalho M Moreau CS. Diversity of *Wolbachia* Associated With the Giant Turtle Ant, Cephalotes Atratus. Curr Microbiol (2019) 76(11):1330–7. doi: 10.1007/s00284-019-01722-8 31254009

[33] Lefoulon E Foster JM Truchon A Carlow CKS Slatko BE. The Wolbachia Symbiont: Here, There and Everywhere. In: Kloc M, editor. Symbiosis: Cellular, Molecular, Medical and Evolutionary Aspects, 1st ed, vol. 69. Cham, Switzerland: Springer (2020), p. 423–51. doi: 10.1007/978-3-030-51849-3 33263882

[34] Shoemaker DD Ross KG Keller L Vargo EL Werren JH. *Wolbachia* Infections in Native and Introduced Populations of Fire Ants (*Solenopsis* Spp.). Insect Mol Biol (2000) 9(6):661–73. doi: 10.1046/j.1365-2583.2000.00233.x 11122476

[35] Tsutsui ND Kauppinen SN Oyafuso AF Grosberg RK. The Distribution and Evolutionary History of *Wolbachia* Infection in Native and Introduced Populations of the Invasive Argentine Ant (*Linepithema Humile*). Mol Ecol (2003) 12:3057–68. doi: 10.1046/j.1365-294X.2003.01979.x 14629385

[36] Enright MC Day NP Davies CE Peacock SJ Spratt BG. Multilocus Sequence Typing for Characterization of Methicillin-Resistant and Methicillin-Susceptible Clones of *Staphylococcus Aureus* . J Clin Microbiol (2000) 38(3):1008–15. doi: 10.1128/JCM.38.3.1008-1015.2000 PMC86325 10698988

[37] Meats E Feil EJ Stringer S Cody AJ Goldstein R Kroll JS. Characterization of Encapsulated and Noncapsulated *Haemophilus Influenzae* and Determination of Phylogenetic Relationships by Multilocus Sequence Typing. J Clin Microbiol (2003) 41(4):1623–36. doi: 10.1128/JCM.41.4.1623-1636.2003 PMC153921 12682154

[38] Elias-Costa AJ Confalonieri VA Lanteri AA Rodriguero MS. Game of Clones: Is *Wolbachia* Inducing Speciation in a Weevil With a Mixed Reproductive Mode? Mol Phylogenet Evol (2019) 133:42–53. doi: 10.1016/J.YMPEV.2018.12.027 30583042

[39] Atyame CM Labbé P Dumas E Milesi P Charlat S Fort P. *Wolbachia* Divergence and the Evolution of Cytoplasmic Incompatibility in *Culex Pipiens* . PloS One (2014) 9(1):e87336. doi: 10.1371/journal.pone.0087336 24498078 PMC3909092

[40] Chifflet L. Guzmán N. V. Rey O. Confalonieri V. A. Calcaterra L. A. (2018). Southern Expansion of the Invasive Ant Wasmannia auropunctata Within Its Native Range and Its Relation With Clonality and Human Activity. PLoS ONE 13(11):L1–16. doi: 10.1371/journal.pone.0206602 PMC6248933 30462663

[41] Dedeine F Ahrens M Calcaterra L Shoemaker DD. Social Parasitism in Fire Ants (*Solenopsis* Spp.): A Potential Mechanism for Interspecies Transfer of Wolbachia. Mol Ecol (2005) 14(5):1543–8. doi: 10.1111/j.1365-294X.2005.02499.x 15813792

[42] Tolley SJA Nonacs P Sapountzis P. *Wolbachia* Horizontal Transmission Events in Ants: What do We Know and What can We Learn? Front Microbiol (2019) 10:296(MAR). doi: 10.3389/fmicb.2019.00296 30894837 PMC6414450

[43] Sánchez-Restrepo AF Chifflet L Confalonieri VA Tsutsui ND Pesquero MA Calcaterra LA. A Species Delimitation Approach to Uncover Cryptic Species in the South American Fire Ant Decapitating Flies (Diptera: Phoridae: *Pseudacteon*). PloS One (2020) 15(7):e0236086. doi: 10.1371/journal.pone.0236086 32678835 PMC7367480

[44] Tseng SP Hsu PW Lee CC Wetterer JK Hugel S Wu LH. Evidence for Common Horizontal Transmission of *Wolbachia* Among Ants and Ant Crickets: Kleptoparasitism Added to the List. Microorganisms (2020) 8(6):1–11. doi: 10.3390/microorganisms8060805 PMC7355411 32471038

[45] Holldobler B Wilson EO. The Ants. Cambridge, Massachusetts, USA: Harvard University Press (1990).

