# Supplementary material for: Wolbachia Infection in Native Populations of the Invasive Tawny Crazy Ant Nylanderia fulva
Source: Front Insect Sci. 2022 Jun 6;2:905803. doi: 10.3389/finsc.2022.905803 (PMC10926365; doi:10.3389/finsc.2022.905803)
Supplement: Supplementary file 1 [file DataSheet_1.pdf]

Wolbachia infection in native populations of the invasive tawny crazy ant _Nylanderia fulva_

Fernández, M. B., Bleidorn, C., and Calcaterra, L. A.

Supplementary material

Figure S1. *Wolbachia* maximum likelihood phylogeny based on *glyQ* gene fragment with host species as labels. Colors highlight the sequences produced in this work belonging to different *Nylanderia* mitochondrial DNA clades: clade I (blue), clade II (yellow), clade III (red), and *Nylanderia* sp. 1 (green). All sequences belong to *Wolbachia* supergroup A unless stated with its corresponding letter (B-F).

Table S1. Ant samples, species names, country and decimal geographic coordinates.

Species	Sample	Country	Latitude	Longitude
Nylanderia fulva (clade I)	N004	Argentina	-25.83337	-54.54778
	N030	Argentina	-33.15833	-59.28369
	N035	Brazil	-28.39980	-54.47698
	N047	Brazil	-25.41841	-52.06957
	N049	Brazil	-25.10950	-52.85403
	N052	Paraguay	-25.48811	-54.79434
	N060	Argentina	-34.57245	-58.51128
	N077	Argentina	-28.62751	-57.41871
	N084	Argentina	-31.73647	-59.91203
	N093	Argentina	-27.72297	-54.91841
	N106	Argentina	-26.97684	-55.11347
	N118	Argentina	-33.82025	-59.51466
	N122	Argentina	-32.92510	-60.75926
	N159	Argentina	-28.01899	-58.02856
N. fulva (clade II)	N001	Argentina	-30.41338	-60.25810
	N007	Argentina	-27.32745	-55.52623
	N018	Brazil	-24.07600	-54.25890
	N034	Uruguay	-30.93441	-55.54810
	N054	Paraguay	-25.25822	-57.71131
	N058	Argentina	-27.44095	-58.89465
	N105	Argentina	-25.68203	-54.44605
	N132	Argentina	-27.11160	-60.58513
N. fulva (clade III)	N002	Argentina	-30.41338	-60.25810
	N010	Argentina	-32.23166	-58.23441
	N022	Argentina	-28.24976	-58.70276
	N031	Argentina	-34.34587	-58.64043
	N059	Argentina	-30.81881	-58.04474

	N070	Argentina	-34.54610	-58.43921
	N091	Argentina	-27.91239	-55.81244
	N125	Argentina	-31.40256	-64.46569
Nylanderia sp. 1	N064	Argentina	-27.28563	-65.87287
	N124	Argentina	-31.72333	-64.40345
Nylanderia sp. 2	N079	Argentina	-28.55425	-57.19963
	N135	Argentina	-34.17489	-58.86293
Paratrechina longicornis	PL150	Argentina	-26.40792	-54.57675

Table S2. Polymerase chain reaction (PCR) protocols for *Wolbachia* MLST system, *wsp* and *glyQ* genes.

PCR Protocol	*Primer* name	Annealing Temperature (°C)	*Primer* sequences (5'-3')
Baldo *et al.* 2006	*fbpA*	56 and 57	Forward (F): GCTGCTCCRCTTGGYWTGAT<br>Reverse (R): CCRCCAGARAAAAYYACTATTC
	ftsZ	51	F: ATYATGGARCATATAAARGATAG<br>R: TCRAGYAATGGATTRGATAT
	glyQ	51	F: GCAATGGAATGGAAGTAACACAG<br>R: YTCACACCAAGCACACCTCT
	wsp	51	F: GTCCAATARSTGATGARGAAAC<br>R: CYGCACCAAYAGYRCTRTAAA
	coxA	54	F: TTGGRGCRATYAACTTTATAG<br>R: CTAAAGACTTTKACRCCAGT
Baldo *et al.* 2006[1] modified protocol:<br>- 94°C for 2 min.<br>- 35 cycles of:<br>94°C for 1 min.<br>Annealing for 1 min.<br>72°C for 1 min.<br>- 72°C for 10 min.	*hcpA*	54	F: GAAATARCAGTTGCTGCAAA<br>R: GAAAGTYRAGCAAGYTCT
Asimakis *et al.* 2019[2]	*gatB*	53 and 58	F: GAKTTAAAYCGYGCAGGBGTT<br>R: TGGYAAYTCRGGYAAAGATGA

Table S1 Footnote:

[1] Baldo, L., Hotopp, J. C. D., Jolley, K. A., Bordenstein, S. R., Biber, S. A., Choudhury, R. R., Hayashi, C., Maiden, M. C. J., Tettelin, H., & Werren, J. H. (2006). Multilocus sequence typing system for the endosymbiont *Wolbachia pipientis*. Appl Environ Microbiol., 72 (11):7098-7110. doi:10.1128/AEM.00731-06

[2] Asimakis, E. D., Doudoumis, V., Hadapad, A. B., Hire, R. S., Batargias, C., Niu, C., Khan, M., Bourtzis, K., & Tsiamis, G. (2019). Detection and characterization of bacterial endosymbionts in Southeast Asian tephritid fruit fly populations. BMC Microbiol., 19 (Suppl 1):1-18. doi:10.1186/s12866-019-1653-x

Table S3. Accession numbers from Genbank and *Wolbachia* MLST database ID's from sequences included in phylogenetic analyses not produced in this paper.

Species (*Wolbachia* strain name)	Genbank	ID
<u>*Nylanderia* phylogeny</u>		
Lasius niger	MT862424.1	
Nylanderia flavipes	NC_049861.1	
Nylanderia fulva	JX426040	
Nylanderia pubens	JX426043	
Nylanderia steinheili	JX426049	
Nylanderia vividula	FJ982466.1	
Zatania albimaculata	BK012214.1	
<u>*Wolbachia* phylogeny</u>		
Aedes albopictus (*w*AlbB)	NZ_RWIK01000001.1	
Anopheles demeilloni (*w*AnD)	NZ_CP084694.1	
Brugia malayi (*w*Bm)	AE017321.1	37
Camponotus textor (Ctex_A)		558
Carposina sasakii (*w*CauA)	NZ_CP041215.1	
Cimex lectularius (*w*Cle)	NZ_AP013028.1	
Ctenocephalides felis (*w*CfeJ)	NZ_CP051157.1	
Diaphorina citri	NZ_CP051266.2	
Drosophila ananassae (strW2.1)	CP042904.1	
Drosophila melanogaster (*w*MelPop)	CP046921.1	
Nasonia oneida (*w*OneA1)	QESS01000003.1	
Onchocerca cervipedis (Ocer_C)		505
Onchocera ochengi (*w*Oo)	HE660029.1	
Paratrechina longicornis (*w*Lon_A, *w*Lon_F)		1827, 1828
Spodoptera picta (Spic_B)	NZ_CP067976.1	
Trichogramma pretiosum (Tpre)		1867
Russell et al. 2008 dataset	EU127553-EU127822	

Table S4. Tests for departure from neutrality for mitochondrial DNA of *Nylanderia fulva*. Tajima's D, Fu and Li's D*, and Fu and Li's F. p>0.1 for all tests.

	N	D	D*	F
Nylanderia fulva (clade I)	12	-1.39161	-1.47177	-1.64869
Nylanderia fulva (clade II)	6	-1.33698	-1.36825	-1.45012

Table S5. MLST sequence types established for *Wolbachia* infected *Nylanderia* spp., *glyQ* allele ID numbers and alternative sequence types when *glyQ* gene is considered (MLST+*glyQ* strains).* sample N084 is coinfected with two *Wolbachia* strains according to MLST data (*w*Nyla8 and *w*Nyla9) and two alleles were found for *glyQ* gene (*glyQ* allele ID's 4 and 5); thus, there are up to four possible MLST+*glyQ* strains, but since we were not able to resolve each strains' combination of alleles, we chose not to overestimate the number of haplotypes and used the lowest estimate, two variants of MLST+*glyQ*: *w*Nyla9 and *w*Nyla10.

Host species (clade)	Sample code	Strain Name (MLST)	*glyQ* allele ID	Strain name (MLST+*glyQ*)
Nylanderia fulva (clade I)	N004	*w*Nyla2	2	*w*Nyla2
	N030	*w*Nyla4	2	*w*Nyla4

	N035	*w*Nyla6	3	*w*Nyla6
	N047	*w*Nyla7	2	*w*Nyla7
	N049	*w*Nyla7	2	*w*Nyla7
	N060	*w*Nyla6	3	*w*Nyla6
	N077	*w*Nyla7	4	*w*Nyla8
	N084*	*w*Nyla8<br>*w*Nyla9	3, 4	*w*Nyla9<br>*w*Nyla10
	N093	*w*Nyla10	2	*w*Nyla11
	N106	*w*Nyla7	2	*w*Nyla7
	N118	*w*Nyla12	4	*w*Nyla13
	N122	*w*Nyla7	4	*w*Nyla8
	N159	*w*Nyla13	3	*w*Nyla14
N. fulva (clade II)	N001	*w*Nyla1	1	*w*Nyla1
	N007	*w*Nyla1	1	*w*Nyla1
	N018	*w*Nyla1	1	*w*Nyla1
	N034	*w*Nyla1	1	*w*Nyla1
	N058	*w*Nyla1	1	*w*Nyla1
	N105	*w*Nyla11	3	*w*Nyla12
	N132	*w*Nyla1	1	*w*Nyla1
N. fulva (clade III)	N022	*w*Nyla3	3	*w*Nyla3
	N031	*w*Nyla5	3	*w*Nyla5
Nylanderia sp. 1	N064	*w*Nyla14	5	*w*Nyla15
	N124	*w*Nyla14	5	*w*Nyla15
