# Supplementary figures and images for: Wolbachia Infection in Native Populations of the Invasive Tawny Crazy Ant Nylanderia fulva
Source: Front Insect Sci. 2022 Jun 6;2:905803. doi: 10.3389/finsc.2022.905803 (PMC10926365; doi:10.3389/finsc.2022.905803)

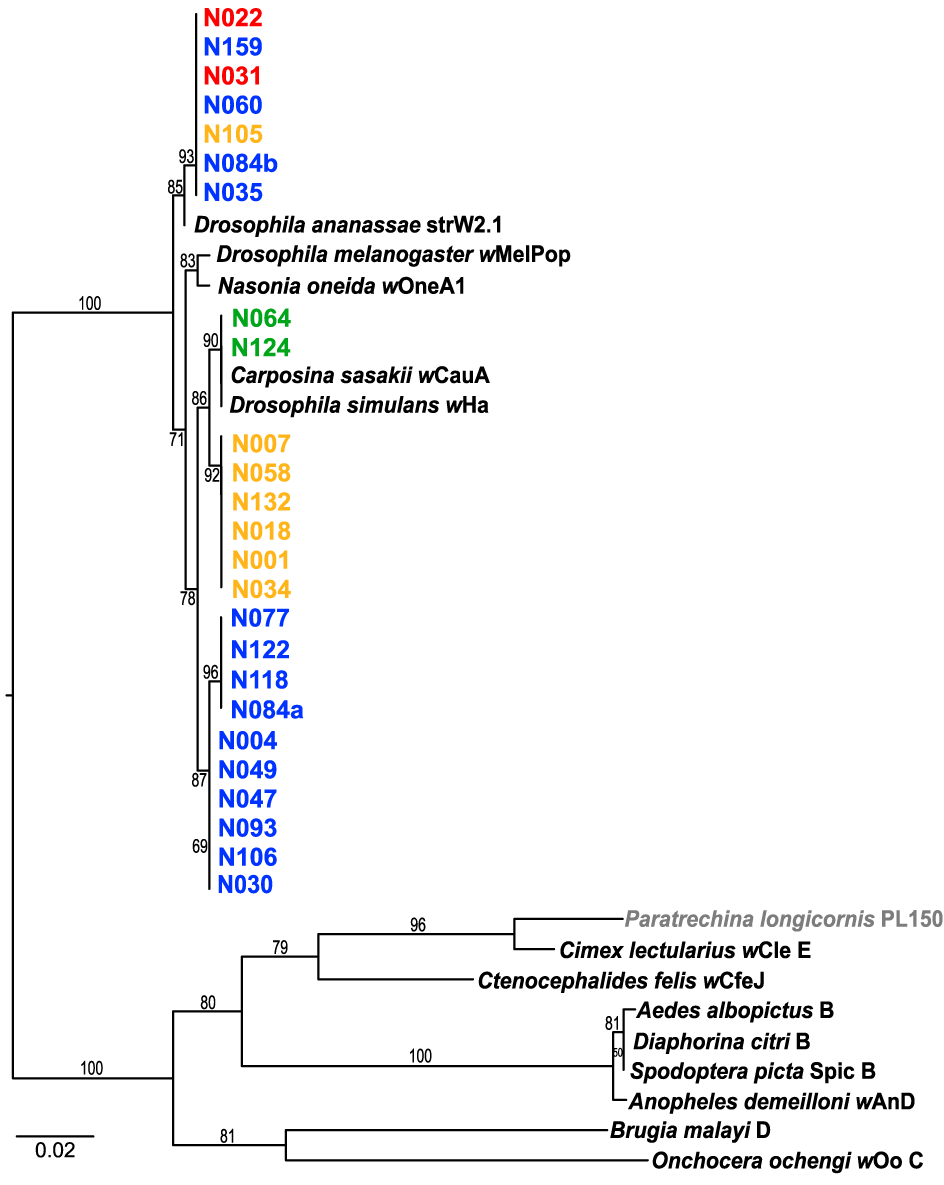

Supplement: Supplementary file 2 [file Image_1.tif]
